# CD32 Expression is not Associated to HIV-DNA content in CD4 cell subsets of individuals with Different Levels of HIV Control

**DOI:** 10.1038/s41598-018-33749-5

**Published:** 2018-10-19

**Authors:** Marcial García, María Angeles Navarrete-Muñoz, José M Ligos, Alfonso Cabello, Clara Restrepo, Juan Carlos López-Bernaldo, Francisco Javier de la Hera, Carlos Barros, María Montoya, Manuel Fernández-Guerrero, Vicente Estrada, Miguel Górgolas, José M Benito, Norma Rallón

**Affiliations:** 1 Instituto de Investigación Sanitaria Fundación Jiménez Díaz, Universidad Autónoma de Madrid (IIS-FJD, UAM), Madrid, Spain; 2 Hospital Universitario Rey Juan Carlos, Móstoles, Spain; 3 Centro Nacional de Investigaciones Cardiovasculares, Madrid, Spain; 4 Hospital Universitario Fundación Jiménez Díaz, Madrid, Spain; 5 Hospital General Universitario Gregorio Marañón, Madrid, Spain; 6 Hospital Universitario de Móstoles, Móstoles, Spain; 7 Hospital Universitario Clínico San Carlos, Madrid, Spain

## Abstract

A recent study has pointed out to CD32a as a potential biomarker of HIV-persistent CD4 cells. We have characterized the level and phenotype of CD32+ cells contained in different subsets of CD4 T-cells and its potential correlation with level of total HIV-DNA in thirty HIV patients (10 typical progressors naïve for cART, 10 cART-suppressed patients, and 10 elite controllers). Total HIV-DNA was quantified in different subsets of CD4 T-cells: Trm and pTfh cells. Level and immunephenotype of CD32+ cells were analyzed in these same subsets by flow cytometry. CD32 expression in Trm and pTfh subsets was similar in the different groups, and there was no significant correlation between the level of total HIV-DNA and the level of CD32 expression in these subsets. However, total HIV-DNA level was correlated with expression of CD127 (rho = −0.46, p = 0.043) and of CCR6 (rho = −0.418, p = 0.027) on CD32+ cells. Our results do not support CD32 as a biomarker of total HIV-DNA content. However, analyzing the expression of certain markers by CD32+ cells could improve the utility of this marker in the clinical setting, prompting the necessity of further studies to both validate our results and to explore the potential utility of certain markers expressed by CD32+ cells.

## Introduction

The existence of HIV reservoirs is the main barrier to HIV eradication^1^. At cellular level HIV latency is mainly found in CD4 T cells with a resting memory phenotype^2–4^, especially in certain subsets such as peripheral follicular T helper cells^5^. Due to the long half life of CD4 subsets harbouring proviral HIV-DNA^6^ as well to additional mechanisms of reservoir maintenance as homeostatic proliferation^7^ or clonal expansion^8^ of latently infected cells, the kinetics of reservoir decline is so slow that purging it with antiretroviral therapy alone is not feasible^6^. Moreover, the different approaches proposed so far have failed in significantly diminishing the size of the HIV reservoir, likely due to different reasons^9,10^.

One of such reasons lies in the difficulty of precisely measuring the HIV reservoir size^11,12^, what has hindered the ability to compare HIV reservoir size in different groups of patients and to estimate the effectiveness of therapeutic approaches aimed to diminish it. Current assays are based either on detection of proviral HIV-DNA content in different types of cells such as peripheral blood mononuclear cells (PBMCs), CD4 cells or subsets of CD4 cells, or on the quantification of virus growth in culture (quantitative viral outgrowth assay, qVOA) that is considered the gold-standard^13^. An inherent drawback of these assays is that since they do not detect HIV at single-cell level, they are not able to identify the phenotype of every single cell carrying latent HIV, what is crucial for our understanding of cell types involved in the maintenance of the reservoir. Although the majority of cellular reservoir resides in CD4 cells with resting memory phenotype, the great majority of cells with this phenotype do not carry HIV. Thus, identifying a cell marker specific for CD4 cells carrying latent HIV would be of great interest not only for the understanding of cellular reservoirs but also as an easily scalable high-throughput assay to precisely measure the *in vivo* frequency of latently infected cells, that could be implemented in clinical trials aimed to purge the HIV reservoir^14^.

In this regard, a very recent paper has pointed to CD32a as a potential biomarker of latently infected CD4 cells^15^. FcγRII (CD32) is a low-affinity cell surface receptor of the immunoglobulin G (IgG) Fc fragment involved in immune response regulation. In human cells three different isoforms have been defined, two activating receptors (CD32a and CD32c), and one inhibitory receptor (CD32b)^16^. CD32 marker is mainly expressed on B-cells, monocytes, granulocytes, platelets and endothelial cells, whereas the expression of this receptor on CD4 T cells is controversial and it seems to be associated to CD4 T cell activation^17–19^. Hovewer, using an *in vitro* infection system, the authors found that CD32a was induced selectively in resting CD4 cells latently infected with HIV but not on those cells actively replicating HIV. Moreover, the content of proviral HIV-DNA was several hundred-fold higher in purified CD4+CD32a+ compared to CD4+CD32a− cells from patients under antiretroviral therapy. From these results the authors conclude that CD32a is a good potential biomarker of persistently infected cells^15^.

To test this hypothesis, in the present study we have characterized the levels and phenotype of CD32+ cells contained in different subsets of CD4 T-cells, and its potential correlation with total HIV-DNA content in two groups of HIV patients with HIV replication control (spontaneously or through cART) and in a group of progressor HIV patients with uncontrolled HIV replication.

## Results

### Characteristics of study population

Table 1 shows the main characteristics of HIV patients enrolled in this study. Briefly, elite controllers (EC), patients with undetectable HIV plasma viremia (pVL) in the absence of antiretroviral therapy (cART) (n = 10), and treated patients (TX) (n = 10), cART-suppressed patients maintaining undetectable pVL, were not significantly different in terms of age, CD4 counts and time since HIV diagnosis (years between the date of HIV diagnosis and the sample collection), although proportion of males was higher in TX compared to EC group. Median length of EC status in EC group was 6 [3–12] years and median length of cART in TX group was 12 [9–16] years. Plasma HIV load and CD4 counts in TP group (typical progressor patients naïve for cART and with high levels of pVL, n = 10) were 4.79[4.46–4.91] log HIV-RNA copies/mL and 599 [518–832] cells/uL respectively. Levels of HIV-DNA in resting memory CD4 T (Trm) and peripheral T follicular helper (pTfh) cells were lowest in EC patients (381 [74–1002] and 88 [73–291] copies/million cells in Trm and pTfh respectively) and highest in TP patients (6768 [3674–12673] and 7737 [4921–9044] copies/million cells in Trm and pTfh respectively), with an intermediate level in TX group (1197[619–1623] and 723 [393–1199] copies/million cells in Trm and pTfh respectively). Moreover, when comparing only EC and TX patients, levels of HIV-DNA in pTfh cells were significantly lower in EC (p = 0.025) and a similar trend was found for Trm cells although the difference was not statistically significant (p = 0.063).

**Table 1:** Characteristics of HIV patients included in the study.

Characteristic	Study Group			p-value^a^	p-value^b^
	EC	TX	TP		
n	10	10	10	—	—
Age (Years)	42 [35–48]	47 [44–51]	37 [31–44]	0.034	0.112
Male (%)	50	90	90	0.003	0.074
Viral load (Log copies/mL)	1.7	1.7	4.79[4.46–4.91]	<0.001	NA
CD4 counts (cells/μL)	872 [634–982]	988 [585–1469]	599 [518–832]	0.354	0.595
Time since HIV diagnosis (Years)	12 [4–14]	13 [11–16]	4.5 [2–5]	0.001	0.234
Lenght of EC status (Years)	6 [3–12]	NA	NA	—	—
Lenght of treatment (Years)	NA	12 [9–16]	NA	—	—
HIV-DNA in Trm cells	381 [74–1002]	1197[619–1623]	6768 [3674–12673]	<0.001	0.063
HIV-DNA in pTfh cells	88 [73–291]	723 [393–1199]	7737 [4921–9044]	0.001	0.025

Data are given as median [IQR], except sex, expressed as %; HIV-DNA content is expressed as copies/million cells; p-value^a^: comparison between the 3 HIV-infected groups (Kruskal-Wallis test); p-value^b^: comparison between EC and TX groups (U-Mann-Whitney test, except sex, evaluated with χ^2^ test); NA: not apply.

### Levels of CD32 expression on different CD4 subsets

Levels of CD32 expression in total CD4, Trm and pTfh cells were analyzed by multiparameter flow cytometry. Supplementary Fig. S1 shows a representative example of CD32 staining in the different groups of subjects analyzed. In all subjects analyzed the population of CD32+ cells consisted mainly of CD32+^dim^ cells and to a much lesser extent of CD32+^bright^ cells. Interestingly, the majority of CD32+^bright^ expressed the activation marker HLADR, except in Trm cells that by definition where HLADR-negative (Supplementary Fig. S1).

**Figure 1:**
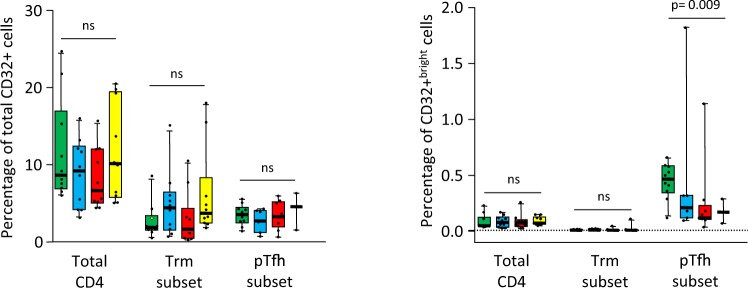
Box-plots showing the levels of CD32+ (dim + bright) (left graph) and of CD32+^bright^ (right graph) cells in different subsets of CD4+ T-cells from uninfected subjects (green boxes), elite controller (blue boxes), cART-suppressed (red boxes) and cART naive (yellow boxes) HIV patients. P-values for the global comparison (Kruskall-Wallis test) are shown. Dotted line in the right graph represents the threshold for detection of CD32+^bright^ cells (0.01% of cells), established using the fluorescence minus one (FMO) control. ns: non significant (p > 0.05).

In the global comparison (Kruskal-Wallis test), levels of total CD32+ cells (dim + bright) in the different CD4 T cells subsets analyzed were not significantly different between the different groups of individuals (Fig. 1, left graph). Overall, levels of total CD32+ cells tended to be higher in TP compared to TX, EC and HC groups in total CD4 T cells (median values: 10.1%, 6.6%, 9.2% and 9.2% respectively) and pTfh cells (median values: 4.6%, 3.3%, 2.7% and 3.5% respectively), although differences were not statistically significant. Similar results were obtained when comparing EC versus TX groups. In Trm cells, EC showed the highest CD32 expression compared to TX, TP and HC groups (median values: 4.4%, 1.6%, 3.7% and 1.9% respectively). Levels of CD32+^bright^ cells were much lower compared to total CD32+ cells, especially in the Trm subset of CD4 cells (Fig. 1, right graph) in agreement with the finding that most CD32+^bright^ expressed the activation marker HLADR (Supplementary Fig. S1). Levels of CD32+^bright^ in total CD4 T cells and in Trm cells were similar in the different groups of subjects analyzed (median values in total CD4 T cells: 0.079%, 0.075%, 0.073% and 0.048%; median values in Trm cells: 0.011%, 0.001%, 0.003% and 0.004% for EC, TX, TP and HC groups respectively), but in pTfh subset, levels of CD32+^bright^ cells were significantly higher in uninfected subjects (0.47%) compared to EC, TX and TP groups (0.21%, 0.12% and 0.17% respectively, p = 0.009) (Fig. 1, right graph).

Intragroup comparisons were performed to ascertain differences in CD32 expression by different subsets of CD4 T-cells (Supplementary Fig. S2). Regarding levels of CD32+ cells, a similar profile was observed in all study groups, with higher levels of CD32 expression in total CD4 cells compared to the rest of CD4 subsets, being the differences statistically significant in all groups of subjects except in TP patients. Interestingly, levels of CD32+^bright^ cells showed a different profile with lower levels in total CD4 cells compared to pTfh subset, and with the lowest levels observed in Trm subset.

### Phenotype of CD32+ cells

Several aspects of CD32+ cells were assessed by multiparameter flow cytometry. Supplementary Fig. S3 shows heat map diagrams showing the differences observed in the level of expression of the different markers analyzed, comparing the different groups of HIV patients with the group of uninfected subjects. CD32+ cells from total CD4 cells of TP patients exhibited several alterations, mainly increased levels of senescence (CD28−CD57+ subset) and activation (HLADR+CD127−subset), increased expression of PD1 marker and decreased expression of CD127 and CXCR3 markers. A similar profile of alterations was observed in CD32+ cells from Trm cells of TP patients. CD32+ from pTfh of TP patients also presented some immune alterations though to a lesser extent. Many of these phenotypic alterations were statistically significant compared to uninfected subjects (Supplementary Fig. S4).

**Figure 2:**
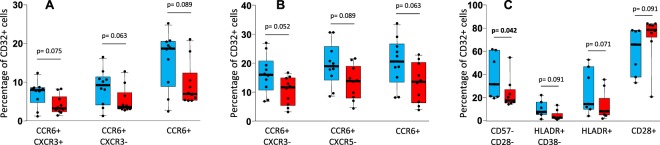
Box-plots showing the levels of different subsets of CD32+ cells from total CD4+ cells (**A**), from Trm cells (**B**), and from pTfh cells (**C**) in elite controller HIV patients (blue boxes), and in cART-suppressed patients (red boxes). P-values for Mann-Whitney U test are shown.

CD32+ cells from HIV patients with suppressed viremia (TX and EC groups) presented lesser immune alterations when compared to uninfected subjects. Overall, CD32+ cells from EC patients presented lower number of alterations than CD32+ cells from TX patients and the profile of alterations was not the same for TX and EC groups with some differences between them. In TX patients, CD32+ cells from total CD4 and from Trm cells presented increased levels of senescence and decreased expression of CD127 and of CCR6, whereas in EC patients the only alterations were an increased expression of PD1 and a decreased expression of CD127 and of CD28. Very few alterations were observed in CD32+ cells from pTfh subset in both TX and EC patients. Some of these alterations were statistically significant compared to uninfected subjects (Supplementary Fig. S4). Lastly, the phenotypic profile of CD32+ cells was compared between EC and TX groups of patients. There were some differences, although most of them were not statistically significant, likely due to the small sample size (Fig. 2). Overall, CD32+ cells from total CD4 and from Trm cells of EC patients expressed higher levels of CCR6 marker, whereas an increased expression of HLADR and a diminished expression of CD28 was observed in CD32+ cells from pTfh cells of EC patients compared to those of TX patients (Fig. 2). Further studies with larger cohorts of patients are needed to corroborate these findings.

The differential expression of several markers in the CD32+ cells from different groups of patients could be associated to the different phenotype of HIV patients included in the study, some with high plasma viral load and others with undetectable viremia, and thus could equally affect both CD32+ and CD32-negative subsets. To address this we analyzed the phenotype of CD32-negative cells and compared with the phenotype of CD32+ cells. Heat map diagrams showing the ratio of the expression level of the different markers between CD32+ and CD32-negative cells (Supplementary Fig. S5) demonstrate clear differences in the phenotypic profile between these two subsets of cells. Independently of the group of subjects (EC, TX, TP or HC), CD32+ cells were more senescent (CD28−CD57+), more exhausted (PD1+) and more activated (HLADR+ and/or CD38+) than CD32-negative cells. In contrast CD32+ cells showed a lower expression of CD28 and CD127 compared to CD32-negative cells. These differences were observed in total CD4 T cells, in Trm and in pTfh cells.

### Correlation between CD32 expression and total HIV-DNA content

In the whole population of HIV patients (EC, TX and TP groups together), we explored the potential correlations between CD32 expression in different subsets of CD4 cells (total CD4 cells, Trm cells and pTfh cells) and the total HIV-DNA levels in Trm and pTfh subsets of CD4 cells. There were no significant correlations (Supplementary Table S6).

However there were several significant correlations between the phenotype of CD32+ cells and total HIV-DNA level in the different subsets of CD4 cells (Supplementary Table S6). Expression of CD127 by CD32+ cells from total CD4 T cells was correlated with HIV-DNA levels in Trm cells; on the other hand, significant correlation between CD127 and CD57 expression on CD32+ cells from total CD4 T cells and total HIV-DNA in pTfh cells was observed. A similar profile was found for the phenotype of CD32+ cells from Trm cells, with CD127 and CD57 markers being the ones that showed the highest correlations with HIV-DNA levels in Trm cells (Supplementary Table S6).

**Figure 3:**
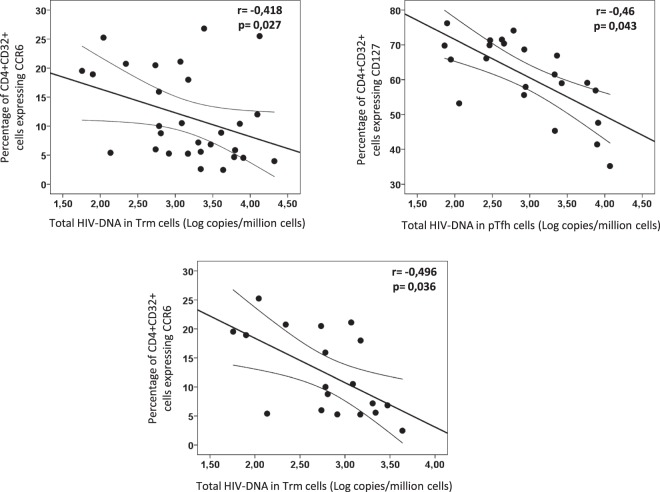
Scatter-plots showing the correlations between phenotype of CD32+ cells and levels of total HIV-DNA in different subsets of CD4 cells, in the whole population of HIV patients (upper row) and in patients with undetectable plasma viremia (lower row). Partial correlation coefficients and p-values after adjusting by type of patient are shown inside the plots.

Since the expression levels of these phenotypic markers varied across the different groups of HIV patients (TP with high level of plasma viremia versus EC and TX with undetectable plasma viremia), a partial correlation coefficient was calculated after adjusting by this factor. After doing this, the majority of significant correlations disappeared (data not shown). The only correlations that remained significant after adjusting by type of patient were the CCR6 expression on CD32 cells from total CD4 cells with HIV-DNA level in Trm cells and the CD127 expression on CD32 cells from total CD4 cells with HIV-DNA level in pTfh cells (Fig. 3). Interestingly, CCR6 expression on CD32-negative CD4 T cells was also significantly correlated with total HIV-DNA level in Trm cells (data not shown), suggesting that CCR6 marker is associated to HIV-DNA content independently of CD32 expression and supporting a more relevant role for CCR6 than for CD32 in the HIV-DNA content of the Trm cells. In contrast to CCR6, no correlation between CD127 expression on CD32-negative CD4 T-cells and total HIV-DNA was observed (data not shown). Thus, the expression of CD127 seems to be associated to total HIV-DNA only in CD32+ cells.

Interestingly, the same analysis only in the groups of patients with undetectable plasma HIV viremia (EC and TX groups) revealed that only the expression of CCR6 marker on CD32+ cells was correlated with levels of HIV-DNA in Trm cells and this correlation remained significant after adjusting by type of patient (Fig. 3). The same was observed for CCR6 in CD32-negative CD4 T-cells (data not shown).

## Discussion

The present study was designed to test the suitability of CD32 molecule as a marker of cells carrying latent HIV and thus as a surrogate measure of HIV-DNA content. For this purpose, we measured levels of CD32 in total CD4 cells and in subsets of CD4 cells carrying persistent HIV in different groups of HIV-infected patients, and checked the potential correlations between CD32 expression and levels of total HIV-DNA in different subsets of CD4 cells. The main findings of our study are: a) There were no significant differences in the level of CD32 expression by CD4 cells between different groups of HIV patients stratified according to the level of HIV viral suppression; b) Levels of CD32 expression did not correlate with levels of total HIV-DNA; c) Expression of certain markers by CD32 cells did correlate with the levels of total HIV-DNA; d) CD32+ cells present a differential phenotype compared to CD32- cells, highlighting the increased expression of HLA-DR what could support that this CD32+ subpopulation is involved in productive HIV infection more than in latent infection.

CD32 has been recently proposed as a potential marker of cells carrying latent HIV^15^ and this has raised great expectations since an easily measurable marker of HIV latency would be an invaluable tool in the fight against HIV reservoirs. Briefly, the findings in the study by *Descours et al*. pointing to CD32 as a potential marker of HIV reservoir were: selective induction of CD32 after infection of resting CD4 cells; several hundred-fold enrichment of HIV-DNA in CD4 cells expressing CD32, especially in the very rare CD32^bright^ population; several thousand-fold enrichment of inducible replication-competent virus in CD32+ cells. However, as the authors point out, not all HIV-DNA was present in CD4 cells expressing CD32 and not all CD32+ cells carried HIV-DNA.

To test the potential utility of CD32 as marker of cells carrying latent HIV, we first compared the levels of CD32+ cells in different subsets of CD4 cells harbouring proviral HIV-DNA and in different groups of HIV patients with different levels of HIV control and of total HIV-DNA levels, under the assumption that CD32 expression will parallel the levels of total HIV-DNA. However, our results demonstrate similar levels of CD32 expression in the different groups of patients analyzed, in spite of the different levels of total HIV-DNA observed in these groups of patients, in agreement with very recently published studies^20,21^. This finding was independent of the CD4 cell subset analyzed (total CD4, Trm or pTfh subsets) and is in agreement with a very recent report showing only very slight differences in the levels of CD32 expression when comparing HIV patients (either viremic or aviremic) and uninfected subjects^22^. Moreover, in our study we were able to clearly differentiate two populations of CD32+ cells in the majority of individuals analyzed: a dim population comprising the great majority of CD32+ cells and a very scarce bright population, in agreement with the study of *Descours et al*.^15^. Interestingly the CD32+^bright^ population presented the highest enrichment in HIV-DNA in the study of *Descours et al*.^15^. Thus we tested if the levels of CD32+^bright^ cells differed among HIV patients according to levels of total HIV-DNA. As for total CD32+ cells, we did not find significant differences between the different groups of patients. Lastly, we compared levels of CD32 in different subsets of CD4 cells and found that CD32 was not preferentially expressed on those subsets where the majority of latently infected cells reside (Trm and pTfh cells), being the expression on total CD4 cells higher than in Trm or pTfh subsets of CD4 cells, suggesting that other subsets of CD4 cells such as naïve cells and/or activated cells express even higher levels of CD32 than CD4 cells with memory phenotype, what is in agreement with a very recent study showing a positive correlation between CD32 expression and activation levels of T cells^22^. Taken together, our results regarding the levels of CD32+ cells do not support CD32 as a surrogate marker of latently HIV infected CD4 cells.

Next, we analyzed the potential correlations between the level of CD32 expression and the levels of total HIV-DNA in different subsets of CD4 cells and found no significant correlations, in agreement with recent reports^20–22^. Only a slight trend of correlation between CD32 expression on pTfh cells and total HIV-DNA levels in this subset was found, that disappeared after adjusting by the type of patient (with or without detectable HIV viremia). This lack of correlation is in accordance with the results regarding the levels of CD32 and reinforces the lack of support to CD32 as a surrogate marker of latently HIV-infected cells in HIV patients with different degrees of viral replication.

We also evaluated the expression of several phenotypic markers by CD32+ cells in order to check for the existence of phenotypic differences between the different groups of HIV patients that could be associated with the levels of total HIV-DNA. CD32+ cells from TP patients presented several phenotypic alterations as compared to cells from uninfected subjects. Overall, CD32+ cells from HIV patients with uncontrolled viral replication (TP) presented a more differentiated, activated and senescent phenotype as well as diminished expression of chemokine receptors CXCR3 and CXCR5. Some but not all of these alterations were normalized in patients with undetectable plasma viremia (TX and EC) and interestingly there were some differences in the phenotypic profile of CD32+ cells between TX and EC patients in spite of similar levels of HIV suppression. These phenotypic differences between groups of patients were specific for CD32+ cells, since the majority of these differences did not exist when analyzing the phenotype of CD32-negative cells, suggesting that the disease phenotype (uncontrolled versus controlled viral replication) does not equally affect the phenotype of CD32+ and CD32-negative subsets of T cells. Moreover, we found some interesting phenotypic differences between CD32+ and CD32-negative cells suggesting that, compared to CD32-negative, CD32+ cells present a characteristic phenotype that could favor productive HIV infection in this cell subpopulation as has been recently suggested^23^.

Of the many phenotypic markers analyzed on CD32+ cells, CD127 and CCR6 were among the markers showing the highest differences between groups of patients. Moreover, CD127 and CCR6 were the only markers that showed a significant correlation with levels of total HIV-DNA after adjusting by patient´s group. Both correlations were inverse and thus lower levels of CD127 or CCR6 expression on CD32+ cells were associated with higher levels of total HIV-DNA, suggesting a role for these markers in HIV reservoir. CD127 is the receptor for IL7, a cytokine with a pivotal role in T-cell homeostasis^24^ and in CD4 memory cells survival and expansion^25^. The role of IL7 in HIV persistence is controversial and previous studies have reported both a latency-reversing effect^26,27^, but also priming for HIV-reservoir maintenance due to the effect of IL7 on survival and expansion of infected CD4 cells^28–30^. The inverse association we found between CD127 expression by CD32+ cells and total HIV-DNA content suggest that cells not expressing CD127, and thus not able to respond to IL7, could have a higher contribution to HIV reservoir. We did not measure total HIV-DNA in cells according to CD127 expression and thus we couldn´t confirm this hypothesis. In contrast to our hypothesis, a previous study reported that the majority of HIV-DNA in CD4 T cells is present in CD127+ cells^31^, although they did not compare HIV-DNA levels between CD127+ with CD127- cells and thus the question is still open.

CCR6 expression was inversely correlated to levels of total HIV-DNA in the whole population of HIV patients. Interestingly this correlation was also observed in HIV patients with undetectable HIV plasma viremia (EC and TX groups together), suggesting a role for this marker in HIV reservoir in patients with complete suppression of viral replication. CCR6 plays an important role in redirecting T cells to gut-associated lymphoid tissue (GALT)^32^ and is considered a marker of Th17 cells^33^. Different findings support a multifaceted role for CCR6 in HIV pathogenesis, contributing to both HIV dissemination and to immunity against HIV^34^. Regarding HIV reservoirs, two recent studies have shown that CCR6+ CXCR3+^35^ or CCR6+^36^ cells are enriched in HIV-DNA, supporting a role for CCR6 in the maintenance of HIV persistence^37^. The inverse association we found between CCR6 and total HIV-DNA seems counterintuitive with these studies. However, other mechanisms not related to differential content of HIV-DNA in CCR6+ versus CCR6- could explain the inverse association. For example, longer survival and proliferation of CCR6+ cells^38^ could dilute the contribution of CCR6-expressing cells to HIV-DNA and this would explain the inverse correlation. Also, given that CCR6 is a marker of Th17 cells, the inverse correlation could simply reflect the fact that those patients with lower total HIV-DNA (EC) are also able to maintain a more preserved mucosal immunity as indicated by a more preserved Th17 population^39^. Lastly, the relatively low correlation coefficient may suggest a spurious correlation biased by other factors not controlled in our study.

There are some controversial issues in our study. First, Descours *et al*. described the selective induction of CD32a after HIV infection of resting CD4 T cells; however, there is no an available antibody specific for CD32a. The only antibody designed to detect the CD32a isoform has shown a very low resolution^40^. The anti-CD32 antibody clone FUN-2 used in our study and in the study by Descours *et al*.^15^ does not distinguish between different CD32 isoforms (CD32a, CD32b and CD32c)^20,23,41^ and thus, we cannot distinguishing them by flow cytometry. Therefore, a particular isoform of CD32 cannot be associated with enrichment in HIV-DNA. Second, the measurement of total HIV-DNA and the absence of virus growth assays to evaluate replication-competent virus in our study could raise concerns; however, several authors recently noted the relevance of measuring total HIV-DNA as a marker of viral reservoir dynamics with clinical implications^42,43^. The authors suggested that both integrated and total HIV-DNA provides an inducible and functional reservoir and predicts *ex vivo* viral outgrowth^43,44^. Additionally, it has been highlighted that the establishment of HIV latency and virus production from unintegrated genomes follows direct infection of resting CD4+ T-cells^45^. Moreover, the use of ddPCR to quantify the total HIV-DNA levels has been associated with greater precision and reproducibility to quantify low HIV-DNA levels compared to other techniques measuring integrated HIV-DNA^46^.

In summary, ours is the first study performing an in-deep characterization of CD32+ cells in HIV patients with different levels of HIV suppression and cell-associated HIV-DNA with the aim to ascertain the suitability of CD32 as a surrogate marker of total HIV-DNA levels. Overall the results of our study do not support the use of CD32 as marker of cells carrying latent HIV and sheds light on the phenotype of CD32+ cells, highlighting important phenotypic differences potentially associated with HIV persistence and prompting the necessity of further studies with larger cohort of HIV patients to both validate our results and to explore the potential utility of certain markers expressed by CD32+ cells that could potentially improve the utility of this marker in the clinical setting.

## Materials and Methods

### Study population

This is a cross-sectional study including patients with chronic HIV-1 infection distributed in three different groups: 10 elite controller (EC group) patients with undetectable HIV plasma viremia (pVL) in the absence of antiretroviral therapy (cART); 10 cART-suppressed patients maintaining undetectable pVL (TX group); and 10 typical progressor patients naïve for cART and with high levels of pVL (TP group). Patients were recruited from four different hospitals in Madrid, Spain, during a period of 12 months. Inclusion criteria for HIV patients have been described elsewhere^47^. A group of 10 uninfected subjects were also included as control group (HC group). The study protocol was approved by the Ethical review board of the Instituto de Investigación Sanitaria-Fundación Jiménez Díaz, Madrid, Spain in accordance with the declaration of Helsinki. All individuals participating in the study signed an informed consent form.

### Purification of resting memory CD4 T (Trm) and peripheral follicular helper T (pTfh) cells

Starting form fresh peripheral blood mononuclear cells (PBMCs), two different subsets of CD4 T-cells were purified: resting memory CD4 T (Trm) cells (defined as CD4+CD45RO+CD25−CD69−HLADR−) and peripheral T follicular helper (pTfh) cells (defined as CD4+ CD45RO+ CXCR5+). For this purpose, immunomagnetic separation was employed as has been described elsewhere^47^. Purity of each CD4 subset was tested by flow cytometry using a Sony Spectral flow cytometer. Median [Interquartile range] purity for the Trm cells in the whole population of subjects analyzed was 95%[93–97%] and for the pTfh cells was 92%[90–93%].

### Measurement of cell-associated HIV DNA

Proviral HIV DNA was quantified in Trm and pTfh cells from each HIV patient. Total cellular DNA was extracted using a QiAmp DNA Mini-Kit following manufacturer instructions. Content of HIV DNA (expressed as copies of HIV-DNA per million cells) was quantified using an ultrasensitive digital droplet PCR (ddPCR) as has been already described^47^.

### Immunophenotypic analysis

Levels of CD32 expression in Trm, pTfh, and total CD4 cells, as well as phenotype of CD32+ cells, were analyzed by multiparametric flow cytometry. For this purpose, three different panels of monoclonal antibodies were designed to stain PBMCs, Trm and pTfh cells respectively. Each panel included 11 different monoclonal antibodies plus a cell viability dye. Using these staining panels, different characteristics of CD32+ cells were analyzed, including senescence, apoptosis, activation, exhaustion, homing potential, response to homeostatic cytokines (CD127 marker, receptor of IL7 cytokine involved in B and T cell development), as well as expression of chemokine receptors CXCR3, CCR6 and CXCR5. A list of monoclonal antibodies included in each staining panel is shown in Supplementary Table S7 and a detailed description of staining and analysis protocol in Supplementary Methods.

### Statistical analysis

The main characteristics of the study groups are expressed as median [interquartile range]. Differences between the different groups of subjects for the different parameters analyzed were tested using non-parametric tests (Kruskall-Wallis, Mann-Whitney U-test and Wilcoxon signed-rank as appropriate). Spearman´s Rho correlation coefficient was used to test the existence of correlations between continuous variables and Chi-square test for the association between categorical variables. All statistical analyses were performed using SPSS software version 15 (SPSS Inc., Chicago, IL, USA). Heat map diagrams were realized using GraphPad Prism version 7 (GraphPad Software, CA, USA). All p-values were two-tailed and were considered significant when lower than 0.05.

## Electronic supplementary material

Supplementary Information

## Data Availability

All data generated or analyzed during this study are included in this published article.
