# Supplementary material for: CD32 Expression is not Associated to HIV-DNA content in CD4 cell subsets of individuals with Different Levels of HIV Control
Source: Sci Rep. 2018 Oct 19;8:15541. doi: 10.1038/s41598-018-33749-5 (PMC6195600; doi:10.1038/s41598-018-33749-5)
Supplement: Supplementary file 1 — Supplementary Information [file 41598_2018_33749_MOESM1_ESM.pdf]

**TITLE PAGE**

# CD32 Expression is not Associated to HIV-DNA content in CD4 cell subsets of individuals with Different Levels of HIV Control

Marcial García[1,2], Maria Angeles Navarrete-Muñoz[1,2], Jose M Ligos[3], Alfonso Cabello[4], Clara Restrepo[1,2], Juan Carlos López-Bernaldo[5], Francisco de la Hera[4], Carlos Barros[6], María Montoya[3], Manuel Fernández-Guerrero[4], Vicente Estrada[7], Miguel Górgolas[4], José M Benito[1,2], Norma Rallón[1,2]

[1]Instituto de Investigación Sanitaria Fundación Jiménez Díaz, Universidad Autónoma de Madrid (IIS-FJD, UAM), Madrid, Spain; [2]Hospital Universitario Rey Juan Carlos, Móstoles, Spain; [3]Centro Nacional de Investigaciones Cardiovasculares, Madrid, Spain; [4]Hospital Universitario Fundación Jiménez Díaz, Madrid, Spain; [5]Hospital General Universitario Gregorio Marañon, Madrid, Spain; [6]Hospital Universitario de Móstoles, Móstoles, Spain; [7]Hospital Universitario Clínico San Carlos, Madrid, Spain;

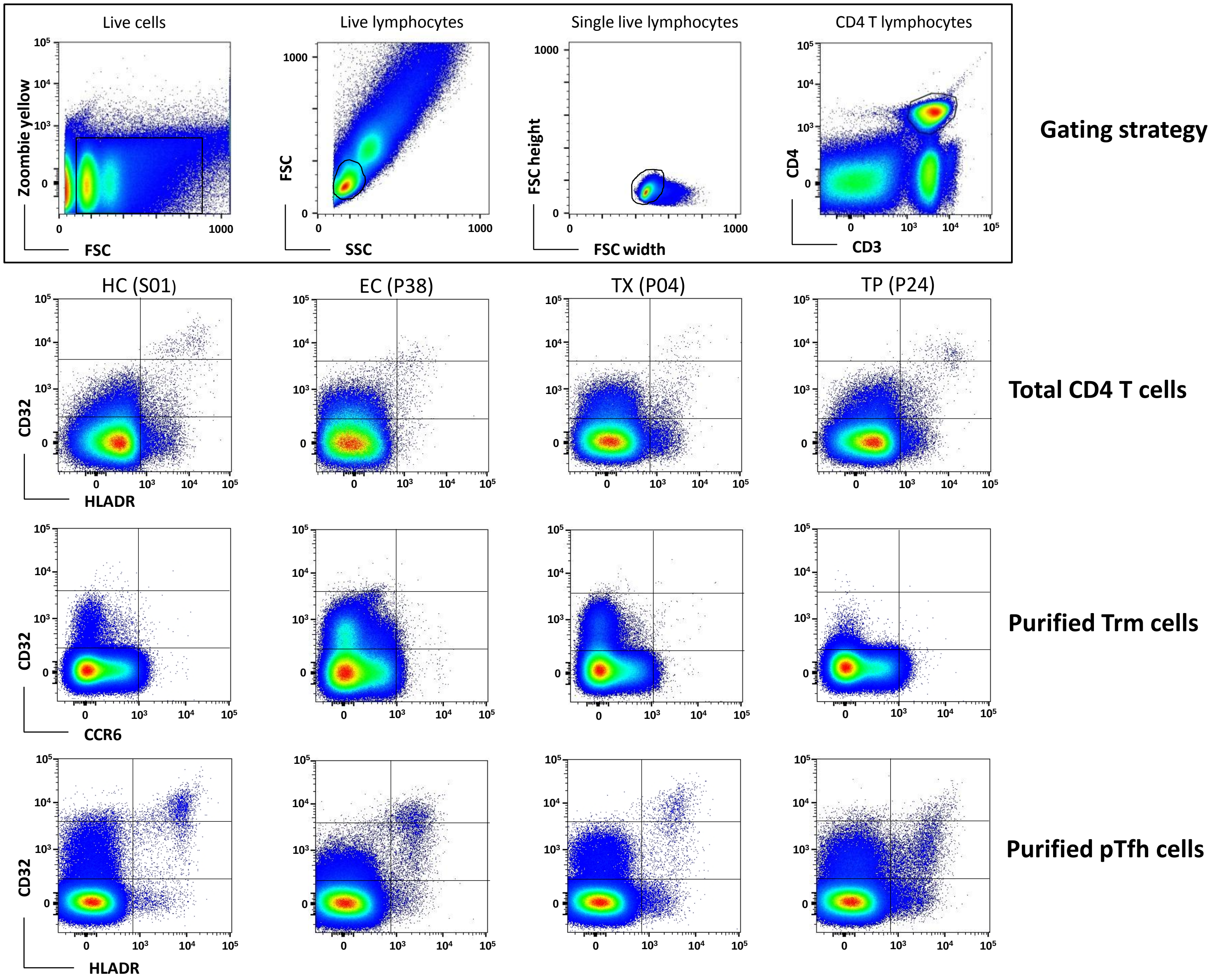


**Supplementary Figure S1**. Representative flow cytometry example of CD32 staining. The upper row shows the strategy of gating for CD4 T cells starting from the whole population of PBMCs. The lower three rows represents CD32 staining in different populations of CD4 T cells (total CD4 T cells gated from PBMCs as shown in the upper row, Trm and pTFH subsets), and each column represents staining in a different group of subjects (HC, EC, TX and TP groups). CD32 versus HLADR dot-plots are shown for total CD4 and pTfh cells; and CD32 versus CCR6 dot-plots are shown for Trm cells. In each dot-plot, CD32+$^{bright}$ cells are in the upper quadrants, CD32+$^{dim}$ cells are in the middle quadrants, and CD32- cells in the lower quadrants.

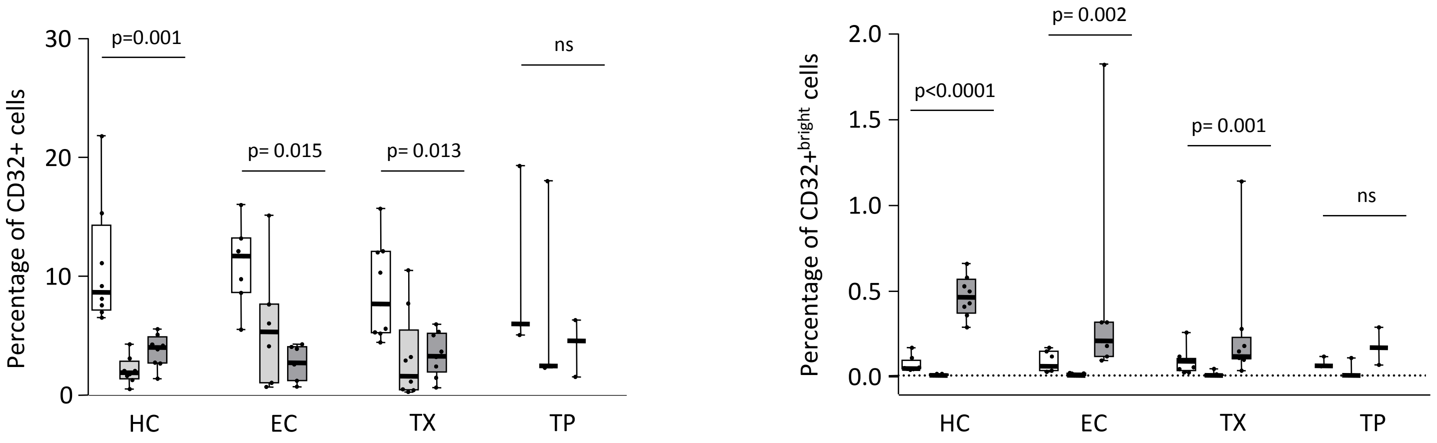


**Supplementary Figure S2**. Box-plots showing the levels of CD32+ (left graph) and of CD32+$^{bright}$ (right graph) cells in total CD4 cells (white boxes), Trm cells (light grey boxes) and pTfh cells (medium grey boxes), in the different groups of individuals included in the study. Dotted line in the right graph represents the treshold for detection of CD32+$^{bright}$ cells (0.01% of cells), established using the fluoresecence minus one (FMO) control. p-values for the global comparison (Friedman test) are shown. ns: non significant (p>0.05).

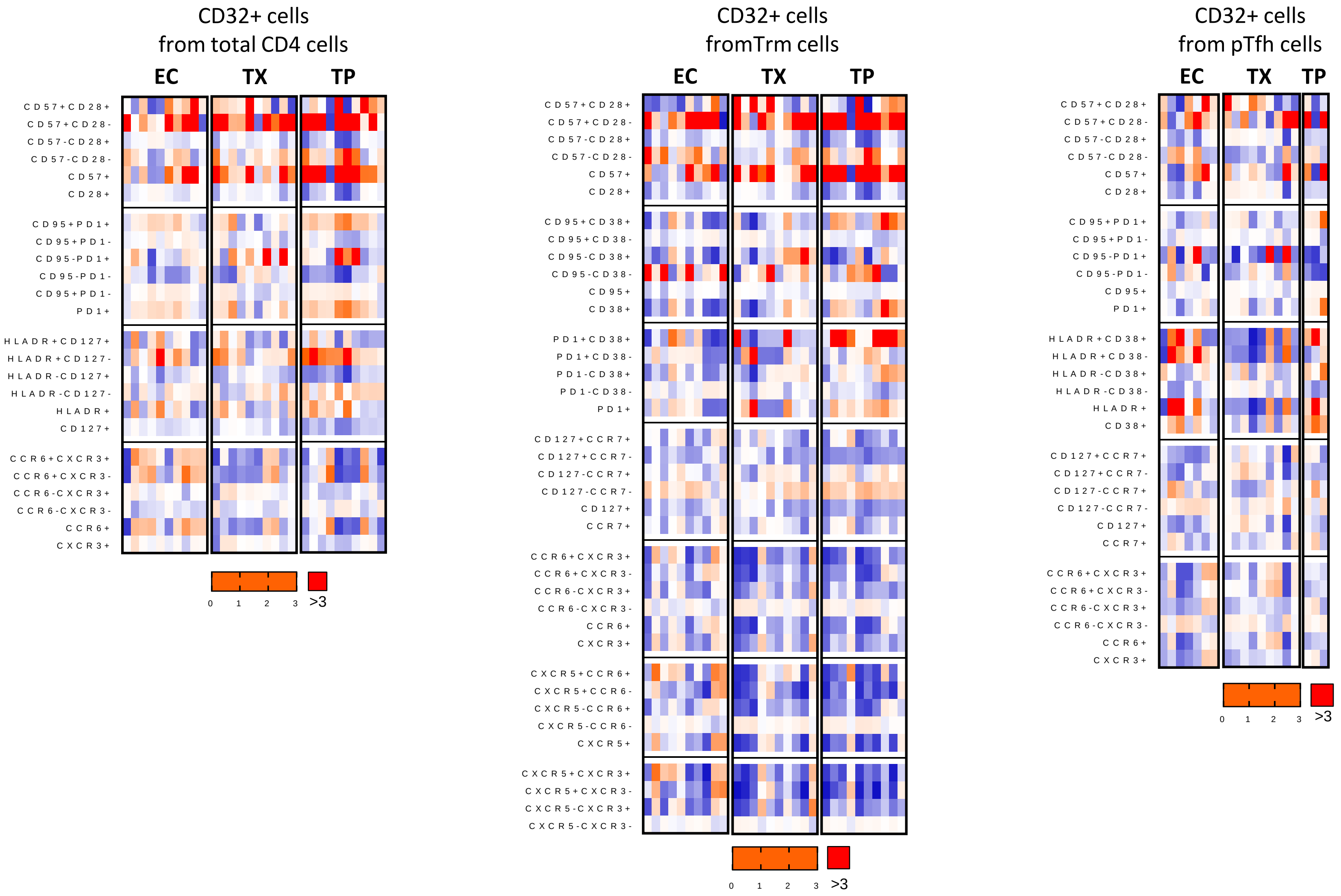


**Supplementary Figure S3**. Heatmap diagrams showing differences in the phenotype of CD32+ cells from different subsets of CD4 T cells (total CD4 T cells, Trm cells and pTfh cells) in different groups of HIV+ patients (EC: elite controller patients; TX: cART-suppressed patiens with undetectable plasma HIV viral load; TP: typical progressor patients naive for cART and high levels of plasma HIV viral load). Different subsets of CD32+ cells are represented in rows and different individuals in columns. Color coding indicates the fold change of each individual value over the median value obtained in the group of uninfected subjects.

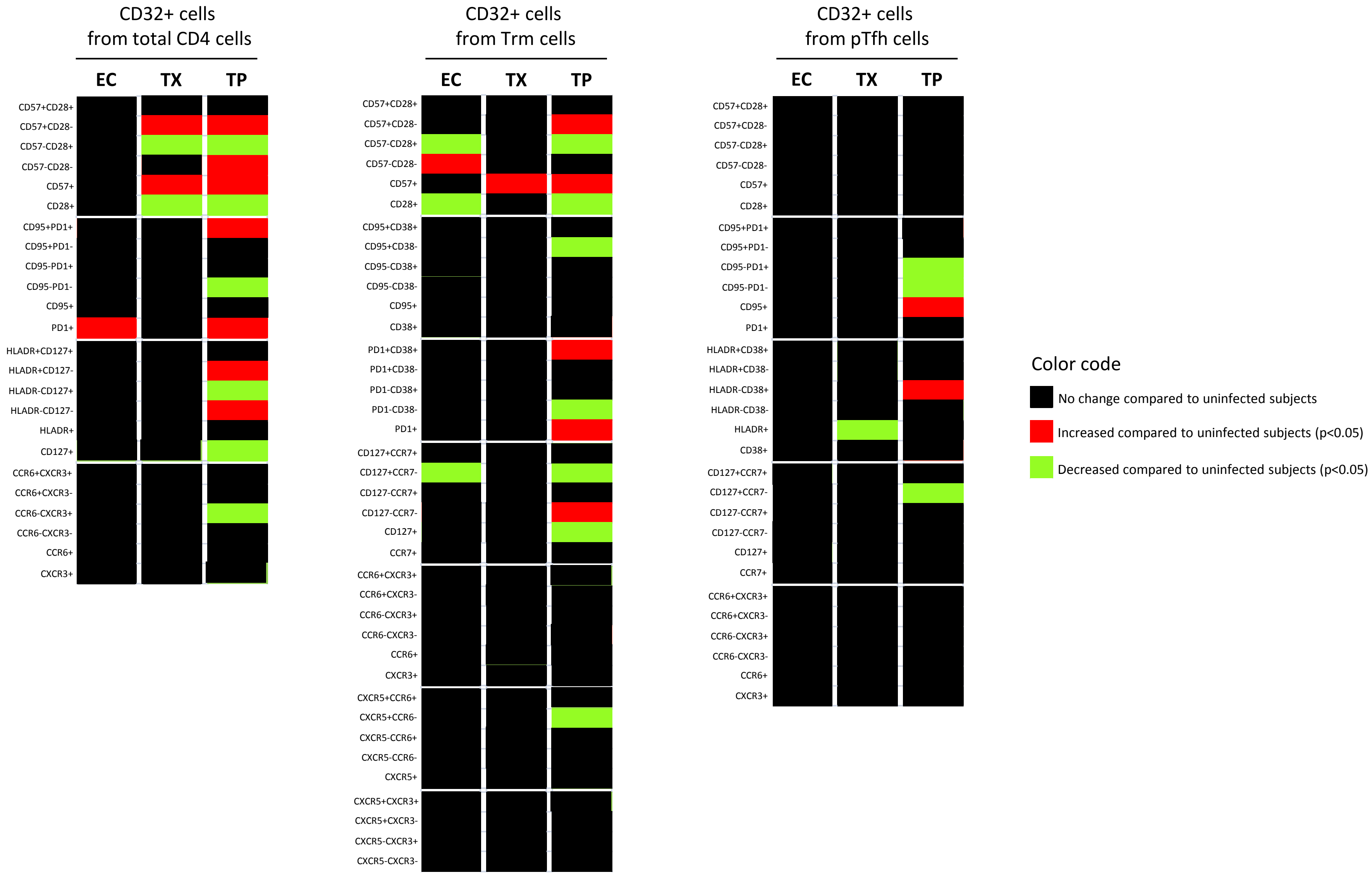


**Supplementary Figure S4**. Color diagram showing statistically significant differences in the phenotype of CD32+ cells from different subsets of CD4 T cells (total CD4 T cells, Trm cells and pTfh cells) of different groups of HIV+ patients (EC: elite controller patients; TX: cART-suppressed patients with undetectable plasma HIV viral load; TP: typical progressor patients naive for cARTand high levels of plasma HIV viral load) compared to uninfected subjects.

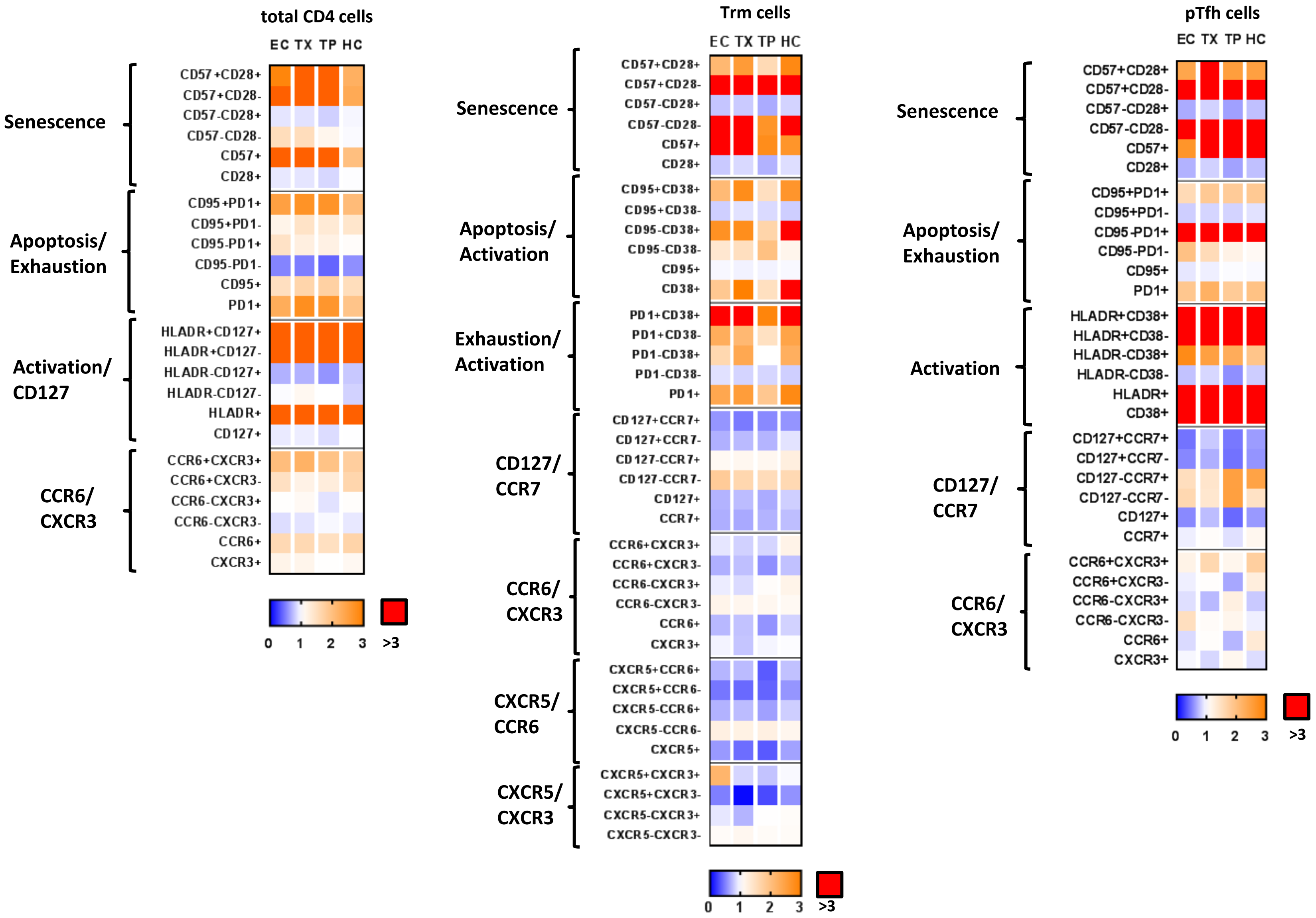


**Supplementary Figure S5**. Heat map diagrams showing phenotypic differences between CD32+ cells and CD32-neg cells from different subsets of CD4 T cells (total CD4 T cells, Trm cells and pTfh cells) of different groups of HIV+ patients (EC: elite controller patients; TX: cART-suppressed patients with undetectable plasma HIV viral load; TP: typical progressor patiens patients naive for cART and high levels of plasma HIV viral load) and uninfected subjects (HC). For each diagram, different subsets defined by the expression of several phenotypic markers are represented in rows and different groups of patients in columns. Color coding indicates the fold change of expression on CD32+ cells compared to the expression on CD32-neg cells.

**Supplementary Table S6**. Spearman´s Rho correlation coefficients between levels of CD32 expression or levels of expression of different phenotypic markers by CD32 cells and HIV-DNA levels in different subsets of CD4 cells in the whole population of HIV patients. Significant (p<0,05) coefficients are in bold.

| VARIABLE | CORRELATION (SPEARMAN´S RHO COEFFICIENT) | | | |
|---|---|---|---|---|
| | HIV-DNA level in Trm cells | p-value | HIV-DNA level in pTfh cells | p-value |
| **Level of CD32 expression on** | | | | |
| Total CD4 cells | 0,296 | 0,119 | 0,111 | 0,622 |
| Trm cells | 0,259 | 0,176 | NA | - |
| pTfh cells | NA | - | 0,41 | 0,091 |
| **Level of expression of different markers on CD32 cells from Total CD4 cells** | | | | |
| CCR6 | -0,361 | 0,054 | -0,344 | 0,117 |
| CXCR3 | -0,335 | 0,076 | -0,417 | 0,053 |
| CD57 | 0,283 | 0,137 | **0,475** | **0,026** |
| CD28 | -0,307 | 0,105 | -0,417 | 0,053 |
| CD127 | **-0,516** | **0,004** | **-0,651** | **0,001** |
| PD1 | 0,279 | 0,143 | 0,398 | 0,067 |
| **Level of expression of different markers on CD32 cells from Trm cells** | | | | |
| CD57 | **0,511** | **0,005** | NA | - |
| CD28 | **-0,380** | **0,042** | NA | - |
| CD127 | **-0,545** | **0,002** | NA | - |
| PD1 | **0,382** | **0,041** | NA | - |
| **Level of expression of different markers on CD32 cells from pTfh cells** | | | | |
| CCR6 | NA | - | -0,416 | 0,086 |

NA: Do not apply

**Supplementary Table S7.** Monoclonal antibodies and fluorochromes included in each staining panel used in the study.

| Antibody | Fluorochrome | Clone | Provider | PBMCs staining panel | Trm staining panel | pTfh staining panel |
|---|---|---|---|---|---|---|
| CD3 | BV650 | UCHT1 | Biolegend | X | - | - |
| CD4 | BV785 | RPA-T4 | Biolegend | X | - | - |
| CD32a | PE | FUN2 | Biolegend | X | X | X |
| CD28 | AF488 | CD28.2 | Biolegend | X | X | X |
| CD57 | PercP-Cy5.5 | HNK-1 | Biolegend | X | X | X |
| CD95 | PE-Cy5 | DX2 | Biolegend | X | X | X |
| CD127 | BV480 | HIL7RM21 | BD Biosciences | X | X | X |
| CD38 | BV785 | HIT2 | Biolegend | - | X | X |
| HLA-DR | BV605 | L243 | Biolegend | X | - | X |
| PD-1 | PE-Dazzle594 | EH12.2H7 | Biolegend | X | X | X |
| CD197 (CCR7) | BV650 | G043H7 | Biolegend | - | X | X |
| CD196 (CCR6) | PE-Cy7 | G034E3 | Biolegend | X | X | X |
| CD183 (CXCR3) | BV711 | G025H7 | Biolegend | X | X | X |
| CD185 (CXCR5) | BV605 | J252D4 | Biolegend | - | X | - |
| - | Zombie yellow BV570 | - | Biolegend | X | X | X |

**Supplementary Methods.** Staining conditions for immunophenotypic analysis and definitions of cell subsets analyzed.

Three different antibody panels were used to stain PBMCs, Trm and pTfh cells. The panel for PBMCs staining included: CD3-B650, CD4-BV785, CD32a-PE, CD28-AF488, CD57-PercPCy5.5, CD95-PECy5, CD127-BV480, HLADR-BV605, PD1-PEDazzle594, CCR6-PECy7, CXCR3-BV711. The panel for Trm cells staining included: CD32a-PE, CD28-AF488, CD57-PercPCy5.5, CD95-PECy5, CD127-BV480, CD38-BV785, PD1-PEDazzle594, CCR7-BV650, CCR6-PECy7, CXCR3-BV711, CXCR5-BV605. The panel for pTf cells staining included: CD32a-PE, CD28-AF488, CD57-PercPCy5.5, CD95-PECy5, CD127-BV480, CD38-BV785, HLADR-BV605, PD1-PEDazzle594, CCR7-BV650, CCR6-PECy7, CXCR3-BV711. All three panels included staining with the viability marker Zombie Yellow BV570.

Two million cells (PBMCs, Trm or pTfh) were washed with phosphate-buffered saline (PBS), resuspended in 100 µL of PBS and stained by incubating with the respective antibody panel for 30 min at 4ºC in the dark. Antibodies conjugated with Brilliant Violet were previously mixed with brilliant stain buffer before being added to the cell samples. Thereafter, cells were washed with PBS and resuspended in 300 µL of PBS for sample acquisition with an SP 6800 Spectral flow cytometer (Sony). Data analysis was done with FlowJo (Treestar, San Carlos, CA). At least 200.000 CD4 T cells, 800.000 Trm cells and 700.000 pTfh cells were collected from each sample for analysis. Taking into account the proportions of CD32+ cells in each subset of CD4 T cells, on average 10.000 CD32+ cells were analyzed for CD4 T, 16.000 CD32+ cells for Trm and 21.000 CD32+ cells for pTfh cells. An initial gating was applied using forward (FSC) and side (SSC) scatter, and then single cells were selected using FSC area versus FSC. Staining

with Zombie Yellow was used to exclude dead cells from analysis. In PBMCs samples, starting from the population of single live cells, a gate was first placed to select CD3+CD4+ cells and then CD32+ cells were gated on CD3+CD4+ cells. In the rest of samples (Trm and pTfh samples), CD32+ cells were directly gated on single live cells because these subpopulations were already purified CD4 T-cells. Thereafter, different parameters were analyzed on CD32a+ cells: senescence (CD28 and CD57 markers), apoptosis (CD95 marker), activation (CD38 and/or HLADR markers), exhaustion (PD1 marker), response to homeostatic cytokines (CD127 marker, the receptor of IL7 cytokine involved in B and T cell development and survival), homing potential (CCR7 marker) and chemokines receptors (CCR6, CXCR3, CXCR5).
